# Effect of arterial oxygen partial pressure inflection point on Venoarterial extracorporeal membrane oxygenation for emergency cardiac support

**DOI:** 10.1186/s13049-021-00902-5

**Published:** 2021-07-08

**Authors:** Hao Zhou, Yi Zhu, Zhongman Zhang, Jinru Lv, Wei Li, Deliang Hu, Xufeng Chen, Yong Mei

**Affiliations:** Emergency Department, Jiangsu Province Hospital and Nanjing Medical University First Affiliated Hospital, Nanjing, 210029 China

**Keywords:** VA-ECMO, tIPPaO_2_, Refractory cardiogenic shock, Cardiac arrest, Cardiac function

## Abstract

**Background:**

Temporary circulatory support is a bridge between acute circulatory failure and definitive treatment or recovery. Currently, venoarterial extracorporeal membrane oxygenation (VA-ECMO) is considered to be one of the effective circulatory support methods, although cardiac function monitoring during the treatment still needs further investigation. Inflection point of arterial oxygen partial pressure (IPPaO2) may occur at an early stage in part of patients with a good prognosis after VA-ECMO treatment, and the relationship between time of IPPaO2 (tIPPaO2) and recovery of cardiac function or prognosis remains unclear.

**Methods:**

To investigate this relationship, we retrospectively analyzed the clinical data of 71 patients with different conditions after treatment with VA-ECMO in the emergency center of Jiangsu Province Hospital between May 2015 and July 2020. Spearman’s correlation analysis was used for the correlation between tIPPaO_2_ and quantitative data, and ROC curve for the predictive effect of tIPPaO_2_ on the 28-day mortality.

**Results:**

Thirty-five patients were admitted because of refractory cardiogenic shock (26 of 35 survived) and the remaining 36 patients due to cardiac arrest (13 of 36 survived). The overall survival rate was 54.9% (39 of 71 survived). Acute physiology and chronic health evaluation II, ECMO time, tIPPaO2, continuous renal replacement therapy time, mechanical ventilation time, and bleeding complications in the survival group were lower than those in the non-survival group, with length of stay, intensive care unit stay, and platelet levels were being higher. The tIPPaO_2_ was negatively correlated with ejection fraction, and the shorter tIPPaO2 resulted in a higher 28-day survival probability, higher predictive value for acute myocardial infarction and fulminant myocarditis.

**Conclusions:**

Therefore, tIPPaO2 could be a reliable qualitative indicator of cardiac function in patients treated with VA-ECMO, which can reveal appropriate timing for adjusting VA-ECMO flow or weaning.

**Trial registration:**

ChiCTR1900026105.

**Supplementary Information:**

The online version contains supplementary material available at 10.1186/s13049-021-00902-5.

## Introduction

Acute circulatory failure (ACF) is a commonly occurring acute severe syndrome in the emergency department. Multiple causes, including myocardial infarction (AMI), fulminant myocarditis (FMC), sepsis-associated cardiomyopathy (SACM), and cardiotoxic drug poisoning, can lead to ACF, which is often manifested as refractory cardiogenic shock (RCS) or even cardiac arrest (CA) [1, 2]. In the last decade, venoarterial extracorporeal membrane oxygenation (VA-ECMO) has been increasingly used for the salvage treatment of ACF because it can be percutaneously performed quickly at bedside, thereby rapidly providing oxygenated blood with a stable flow rate for cardiopulmonary replacement until definitive treatment or recovery of cardiac function [1–5].

However, due to lack of randomized controlled trial (RCT), many problems associated with VA-ECMO still need further exploration, such as monitoring of cardiac function during treatment and overall prognosis [1, 5]. The oxygenated blood flow pumped by peripheral VA-ECMO is mostly opposite to that pumped by the heart. The higher reversed blood flow rate may generate a series of hemodynamic changes to delay recovery of cardiac function [5]. Therefore, it is essential to closely monitor cardiac function and timely adjust VA-ECMO blood flow according to the state of cardiac function.

**Fig. 1:**
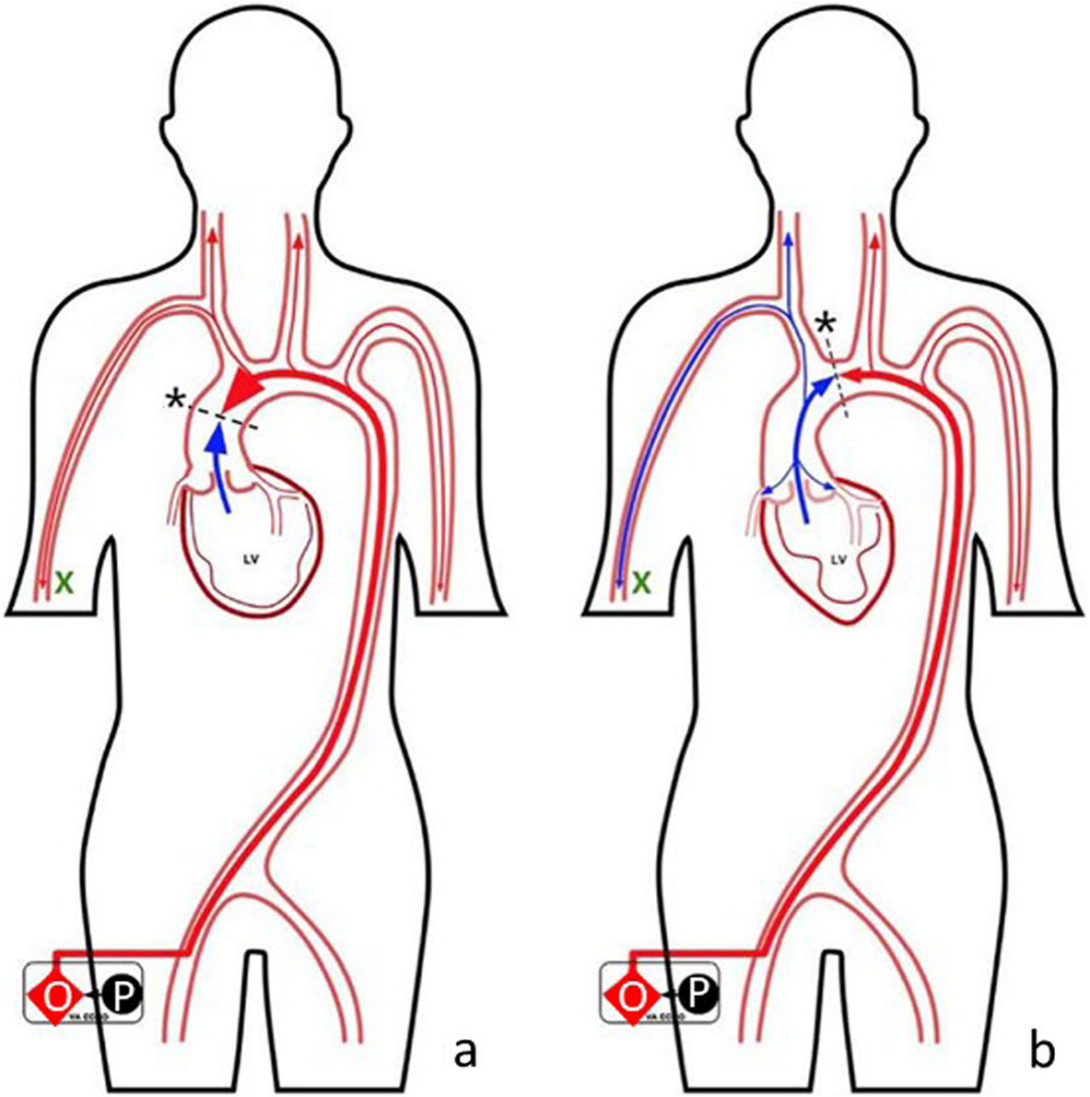
Schematic diagram of the retrograde blood flow from VA-ECMO against the antegrade blood flow pumped by the heart. Legend: The red diamond stands for the ECMO oxygenator, LV for the left ventricle, red vessels for arteries, red lines and arrows for the direction of ECMO oxygenated blood flow, and the blue lines and arrows for the direction of the pumping blood flow from the heart. The black dotted line represents the intersection plane of blood flow, and the black * indicates the IPPaO_2_. The intersection plane is located in the front of the opening of the brachiocephalic trunk when the cardiac function is poor (**a**); When the cardiac function gradually improves, the self-pumping blood flow rate increases, and the intersection plane moves to the distal part of the brachiocephalic trunk opening (**b**).

The area where VA-ECMO retrograde blood flow mixes with antegrade blood flow pumped by the heart is called a “watershed” [5, 6]. In our experience, it has been found that the “watershed” is located in the front of the opening of the brachiocephalic trunk when the cardiac function is poor, while arterial oxygen partial pressure (PaO_2_) measured using the right upper brachial artery catheterization is dominated by the ECMO flow. At this time the PaO_2_ can reach the level of 300 mmHg (Fig. 1a). As the cardiac function gradually improves, the self-pumping blood flow rate increases and the “watershed” moves to the distally of the brachiocephalic trunk opening. The PaO_2_is mainly the cardiac blood flow when PaO_2_ is low at about 100 mmHg (Fig. 1b). Therefore, a sudden decrease in PaO_2_ in the right upper arm and the occurrence of the “inflection point” change may be closely related to cardiac function. The primary objective of this retrospective study was to determine the association between time of inflection point of PaO2 (tIPPaO2) and cardiac function in patients with different diseases. In addition, we also sought to evaluate the association between tIPPaO2 and overall prognosis.

## Materials and methods

### Study design

The study was approved by the Ethics Committee of the First Affiliated Hospital of Nanjing Medical University (Jiangsu Province Hospital). Informed consent was signed by legal representatives of all patients before catheterization. Clinical data of 92 patients diagnosed with ACF requiring VA-ECMO support treatment at the emergency center of the hospital between May 2015 and July 2020 were retrospectively analyzed. The data included patient characteristics, diagnosis and supportive ACF treatment, treatment monitoring, and prognosis (28-day survival rate, intensive care unit (ICU) stay, length of stay, continuous renal replacement therapy (CRRT) time, and ejection fraction (EF) value at discharge).

### Patient selection

Inclusion criteria: patients treated with VA-ECMO for CA or RCS with a treatment duration of ≥72 h. CA initiation criteria: 1) in-hospital cardiac arrest; 2) out-of-hospital cardiac arrest with witnesses and effective cardiopulmonary resuscitation (CPR) within 10 min; 3) age of ≤65 years; 4) suspected reversible causes; and 5) CPR time before initiation of ≤60 min [7]. RCS initiation criteria: 1) persistent hypotension after traditional treatment, including full fluid resuscitation and high dose vasopressors; 2) blood lactate (≥4 mmol/L); 3) evidence of organ hypoperfusion; and 4) bedside echocardiography suggesting ventricular ejection dysfunction. Exclusion criteria: 1) past cardiac disease affected cardiac EF; 2) post-cardiac surgery; 3) mode conversion during ECMO; 4) treatment time of ≤72 h; and 5) VA-ECMO with non-femoral artery-vein catheterization [4, 8].

### Supportive treatment

Supportive treatment: 1) ECMO: all patients were in the peripheral VA mode of femoral artery-vein catheterization, and the initial blood flow maintained mean arterial pressure at 65 mmHg; the ratio of air flow to blood flow was 1:1 with an oxygen concentration of 100%, which then increased or decreased according to the arterial blood gas analysis result; 2) mechanical ventilation (MV): mechanical ventilation after endotracheal intubation with synchronous intermittent mandatory ventilation, tidal volume of 8–10 mL/kg, respiratory rate of 12–20 times/min, inhaled oxygen concentration of 40–60%, and positive end-expiratory pressure of 5–10 cmH_2_O; and 3) continuous renal replacement therapy (CRRT): renal function monitoring, and CRRT treatment if continuous oliguria (urine volume < 0.5 mL/(kg·h)), severe electrolyte disturbance, or progressive elevation of creatinine and urea nitrogen occurred.

### Treatment monitoring

Treatment monitoring was performed using catheterization of the right radial or brachial artery, arterial blood pressure monitoring, observing pulse pressure difference and arterial waveform in real-time, analyzing arterial blood gas and activated clotting time (ACT) every 4–6 h (Fig. 1, green fork represents monitoring point), and performing bedside cardiac ultrasound every 24 h. If the pulse pressure difference suddenly became large or the amplitude of the arterial waveform suddenly increased, blood gas analysis was performed immediately [9, 10]. If there was an inflection point change in PaO_2_, tIPPaO_2_ was recorded and bedside cardiac ultrasound was performed immediately.

### Statistical analysis

SPSS 24.0 (IBM Corp., Armonk, NY, USA) was used for statistical analysis. Quantitative data meeting normal distribution were expressed as mean ± standard deviation. Independent sample t-test was used to compare the differences between groups. Median (quartile) was used to describe the distribution of quantitative data with a non-normal distribution. Mann Whitney U test was used to compare the differences between groups. Frequency and constituent ratio were used to describe the distribution of qualitative data. Chi square test or Fisher’s exact probability method was used to compare the differences between groups. Spearman’s correlation analysis was done for the correlation between tIPPaO_2_ and quantitative data, while ROC curve was used to analyze the predictive effect of tIPPaO_2_ on the 28-day mortality. *P* value of < 0.05 was regarded as statistically significant.

## Results

### Patient characteristics

**Fig. 2:**
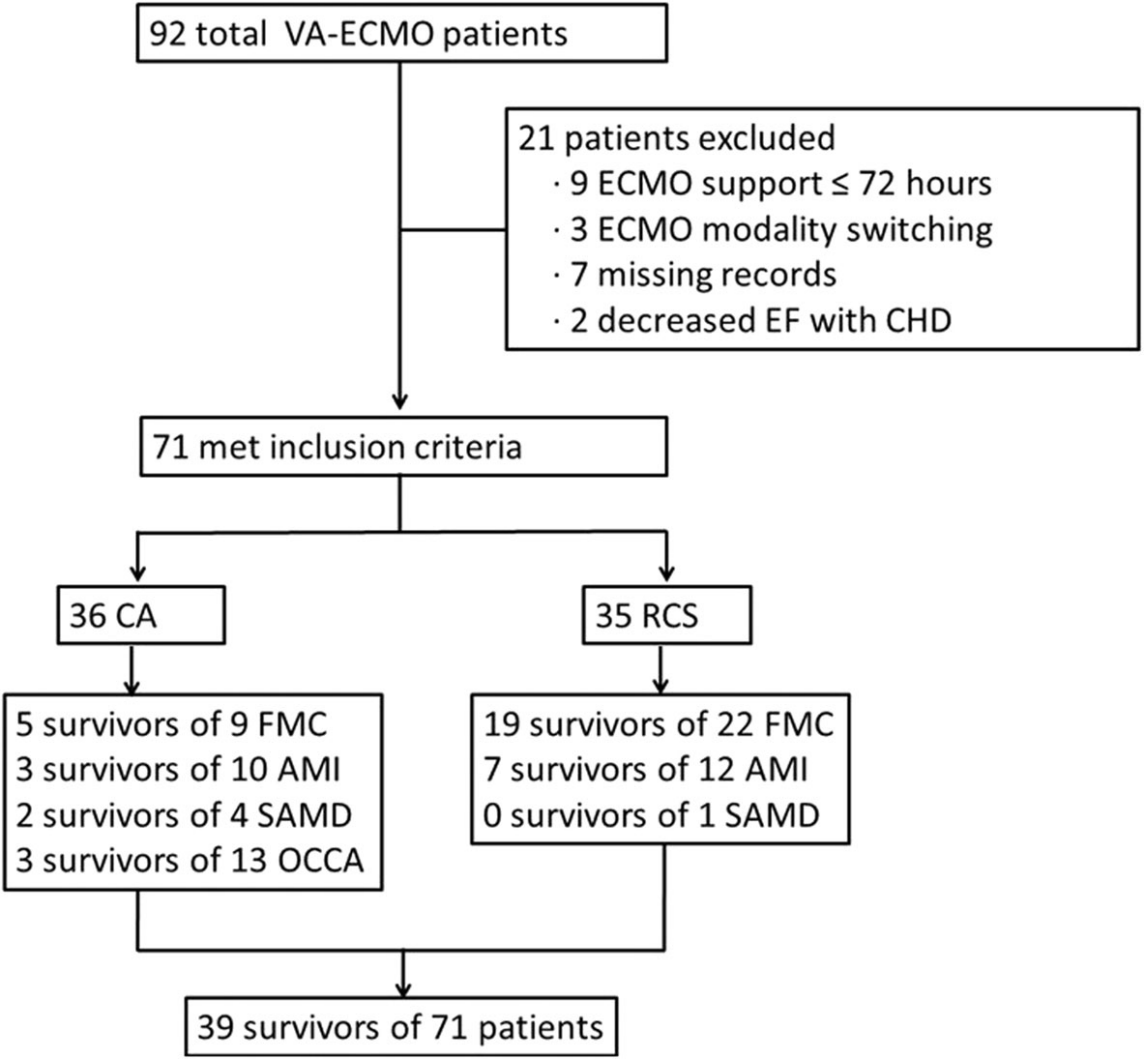
Summary of treatment for CA and RCS patients with VA-ECMO. Legend: VA-ECMO, venoarterial extracorporeal membrane oxygenation; CHD: chronic cardiac dysfunction; RSC, refractory cardiogenic shock; CA, cardiac arrest; EF, ejection fraction; AMI, acute myocardial infarction; FMC, fulminant myocarditis; SACM, sepsis-associated cardiomyopathy; OCCA, other causes related cardiac arrest

We collected the clinical data of 92 patients, of which 21 were excluded because their ECMO support time was ≤72 h (*n* = 9), mode changed from VA to VAV during ECMO (*n* = 3), data were incomplete (*n* = 7), and past cardiac disease affected EF (*n* = 2). A total of 71 patients were included in the final statistical analysis (Fig. 2). Totally 39 of the 71 patients (54.9%) survived, of which 35 were treated with VA-ECMO for RSC (26 survivors of 35 patients) and the remaining 36 were treated with CA (13 survivors of 36 patients). The most common primary disease was FMC (24 survivors of 31 patients), followed by AMI (10 survivors of 22 patients), SACM (2 survivors of 5 patients), and other causes related cardiac arrest (OCCA, 3 survivors of 13 patients), including two patients with trauma-related cardiac arrest (1 survivors of 2 patients), three with pulmonary embolism (2 survivors of 3 patients), four with poisoning-associated myocardial depression, one with hyperthyroidism, one with ketoacidosis, one with severe electrolyte disorder, and one with CA during puerperium.

The average participant age was 44.8 ± 16.7 years and 63.4% of the patients were male (*n* = 45). There were no significant differences in age, sex, body mass index (BMI), comorbidities (hypertension, diabetes, past definite history of coronary heart disease, history of arrhythmia, chronic lung disease, autoimmune diseases, and tumors), history of tobacco and alcohol use, or catheter-related blood-borne infections between the survival and non-survival groups. The median acute physiology and chronic health evaluation (APACHE) II score in the survival group was significantly lower than that in the non-survival group (24.0 vs. 34.5). The ECMO support time (131.0 vs. 168.5 h), tIPPaO_2_ (30.0 vs. 92.0 h), CRRT time (0.0 vs. 6.0 h), and MV time (7.0 vs. 9.0 h) were significantly lower in the survival group than in the non-survival group, while the length of stay (20.0 vs. 9.0d) and ICU stay (17.0 vs. 9.0d) were significantly longer than those in the non-survival group. The minimum platelet count in the survival group was significantly higher than that in the non-survival group (69.5 vs. 31.0*10^9/L), while bleeding complications were less than those in the non-survival group (3 vs. 16 cases; Table 1).

**Table 1:** Baseline characteristics of patients on VA-ECMO for ACF

	Survivor	No- Survivor	*p*-value
Age	43.9 ± 16.7	45.8 ± 16.8	0.638
Sex(M:F)	22/17	23/9	0.220
BMI	23.1 ± 3.4	24.1 ± 3.5	0.227
Comorbidities, n (%)			
Coronary artery disease	1 (2.6)	1 (3.1)	> 0.999
Hypertension	5 (12.8)	8 (25.0)	0.227
Diabetes Mellitus	3 (7.7)	7 (21.9)	0.168
Lung disease	1 (2.6)	2 (6.3)	0.585
Previous Arrhythmia	0 (0.00)	2 (6.3)	0.200
Tumour	3 (7.7)	2 (6.3)	> 0.999
Autoimmune disease	3 (7.7)	0 (0.00)	0.247
Smoking	10 (25.6)	11 (34.4)	0.446
Alcohol	9 (23.1)	5 (15.6)	0.553
APACHE II	24.0 [19.0, 31.5]	34.50 [28.3, 36.0]	0.002
Protopathy			0.003
FMC	24 (61.5)	7 (21.9)	
AMI	10 (25.6)	12 (37.5)	
SACM	2 (5.1)	3 (9.4)	
OCCA	3 (7.7)	10 (31.3)	
Reason of ECMO			0.002
RSC	26 (66.7)	9 (28.1)	
CA	13 (33.3)	23 (71.9)	
Complications			
Bleeding	3 (7.7)	16 (50.0)	< 0.001
CRBSI	0 (0.0)	3 (9.4)	0.087
ECMO support time	131.0 [116.0, 178.0]	168.5 [124.3, 219.3]	0.047
tIPPaO2	30.0 [17.5, 50.0]	92.00 [48.8, 148.5]	< 0.001
ICU time	17.0 [15.0, 24.5]	9.0 [7.0, 13.3]	< 0.001
length of stay	20.0 [17.5, 30.0]	9.00 [7.0, 13.3]	< 0.001
CRRT time	0.0 [0.0, 5.0]	6.0 [3.0, 8.3]	0.001
MV time	7.0 [5.0, 9.0]	9.00 [7.0, 12.3]	0.022
Platelet	69.5 [50.3, 93.0]	31.0 [22.8, 42.3]	< 0.001

*BMI* body mass index, *APACHE* acute physiology and chronic health evaluation, *FMC* fulminant myocarditis, *AMI* acute myocardial infarction, *SACM* sepsis-associated cardiomyopathy, *OCCA* other causes related cardiac arrest, *RSC* refractory cardiogenic shock, *CA* cardiac arrest, *CRBSI* catheter-related blood stream infection, *tIPPaO2* time of inflection point of arterial oxygen partial pressure, *ICU* intensive care unit, *CRRT* continuous renal replacement therapy and *MV* mechanical ventilation

### Predicting 28-day mortality using tIPPaO_2_

**Fig. 3:**
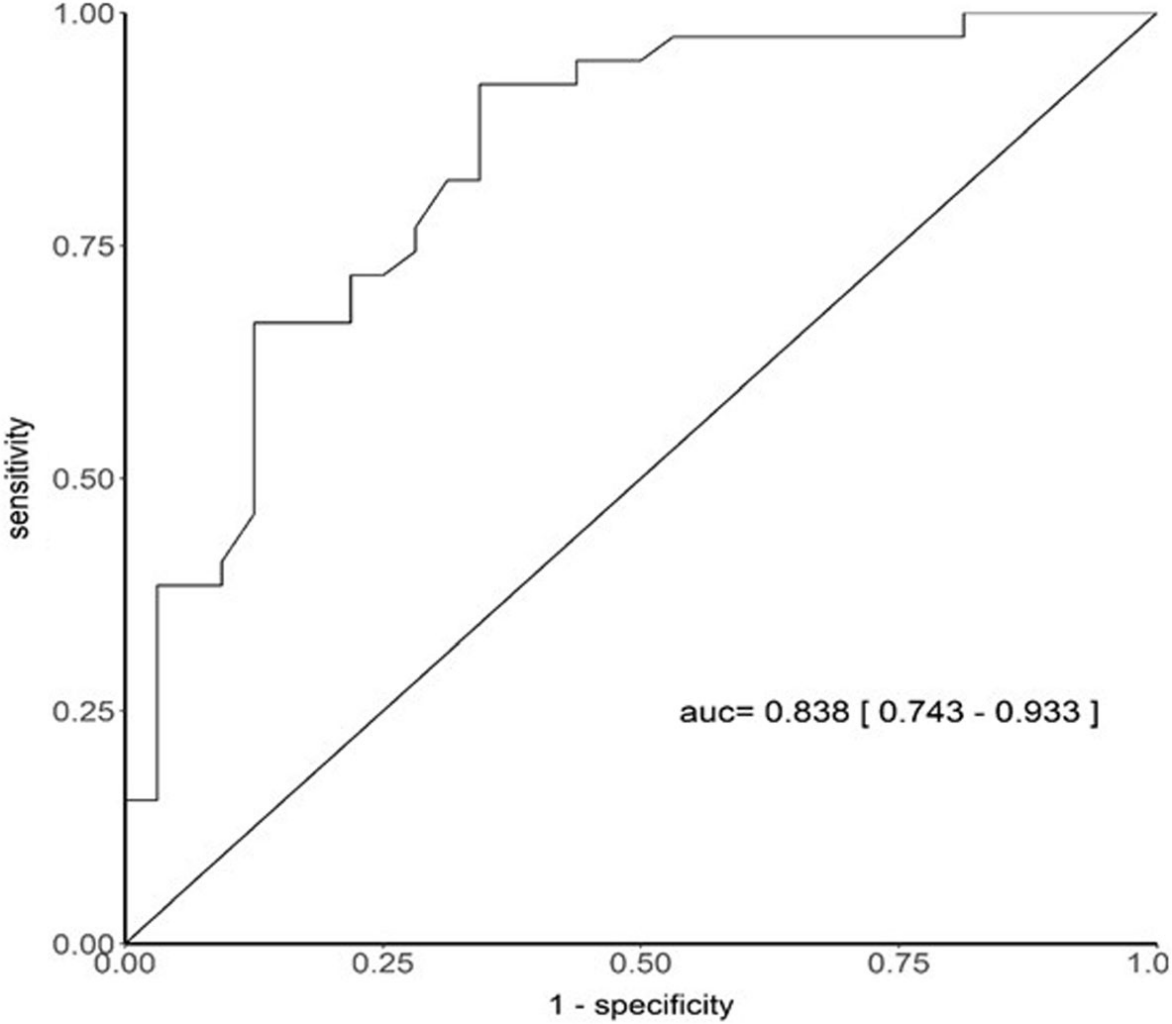
The ROC curve to predict 28-day mortality using tIPPaO2. Legend: The cut-off value was set at 62 h, prediction sensitivity was 92.3%, and specificity was 65.6%. tIPPaO2, time of inflection point of arterial oxygen partial pressure

Mann-Whitney U test analysis showed that tIPPaO_2_ was significantly shorter in the survival group than that in the non-survival group. The ROC curve demonstrated that as the tIPPaO_2_ decreased_,_ the 28-day survival probability increased. The cut-off value was set at 62 h, prediction sensitivity was 92.3%, and specificity was 65.6% (Fig. 3). The ROC curve constructed for different diseases (FMC, AMI, SACM, and OCCA) identified that the tIPPaO_2_ cut-off value for FMC was 68 h, with the best sensitivity and specificity of 100 and 85.7%, respectively. This was followed by the tIPPaO_2_ cut-off value of 83.5 h for AMI, with sensitivity and specificity of 100 and 50.0%, respectively. The tIPPaO_2_ had no predictive value for SACM and OCCA (Table 2).

**Table 2:** Predicting 28-day mortality using tIPPaO2 for different diseases

	Group	AUC	P	Cutoff	Sensitivity	Specificity
Protopathy	FMC	0.92 (0.786–1.000)	< 0.001	68.0	100.0	85.7
	AMI	0.767 (0.561–0.972)	0.011	83.5	100.0	50.0
	SACM	0.667 (0.000–1.000)	0.655	76.0	50.0	66.7
	OCCA	0.633 (0.089–1.000)	0.631	37.0	66.7	90.0

*FMC* fulminant myocarditis, *AMI* acute myocardial infarction, *SACM* sepsis-associated cardiomyopathy and *OCCA* other causes related cardiac arrest

### Correlation between tIPPaO2 and EF value, invasive treatment time, and relevant laboratory tests

Spearman’s correlation analysis was used to study the correlation between tIPPaO_2_ and recent EF value, long-term EF value, invasive treatment time, and relevant laboratory tests. It was found that tIPPaO_2_ was negatively correlated with post-inflection EF, pre-discharge EF, ICU stay, length of stay, and PLT. It was positively correlated with CRRT treatment time. The tIPPaO_2_ had no correlation with peak TnT, peak BNP, duration of ECMO support time and time of MV (Table 3).

**Table 3:** Correlation between tIPPaO2 and EF value, invasive treatment time, and relevant laboratory tests

Factors	tIPPaO2	
	r	P
Post-inflection EF	−0.528	0.000
Pre-discharge EF	−0.546	0.000
ICU stay	−0.404	0.003
Length of stay	−0.426	0.001
Time of ECMO	0.208	0.327
Time of MV	0.199	0.327
Time of CRRT	0.319	0.033
Platelet	−0.457	0.001
Peak TnT	0.142	0.474
Peak BNP	0.025	0.835

*EF* ejection fraction, *ICU* intensive care unit, *CRRT* continuous renal replacement therapy, *MV* mechanical ventilation, *TnT* Troponin T and *BNP* brain natriuretic peptide

The correlation analysis between the tIPPaO_2_ and recent EF in different diseases showed that tIPPaO_2_ was significantly negatively correlated with recent EF in patients with FMC and AMI. There was no correlation between IPPaO_2_ and recent EF in patients with SACM and OCCA (Table 4).

**Table 4:** Correlation between the tIPPaO2 and Post-inflection EF in different diseases

Protopathy	r	P
FMC	−0.541	0.002
AMI	−0.487	0.021
SACM	−0.100	0.873
OCCA	−0.247	0.415

*FMC* fulminant myocarditis, *AMI* acute myocardial infarction, *SACM* sepsis-associated cardiomyopathy and *OCCA* other causesrelated cardiac arrest

## Discussion

VA-ECMO arterial blood flow is reversed compared to the cardiac pump blood flow. High level of retrograde blood flow may enhance cardiac afterload, increase myocardial work, and affect cardiac recovery. Increased afterload can also lead to elevated left ventricular end-diastolic pressure, possibly worsening pulmonary edema [5]. Therefore, timely monitoring of cardiac function and adjustment of VA-ECMO flow are essential for the recovery of cardiac function in ACF patients. It has been reported that the location of the “watershed” is closely related to the cardiac function, which can indirectly reflect the balance between cardiac function and VA-ECMO retrograde flow. Aortic computed tomography angiography and aortic contrast-enhanced ultrasound can accurately reflect the location of the “watershed”, although it is difficult to realize real-time monitoring [9]. The present study found that an abrupt decrease in PaO_2_ (inflection point) monitored using right radial/right brachial artery catheterization can indicate that the “watershed” moved from the proximal end of the brachiocephalic trunk to the distal end, representing a “trigger” improvement of cardiac function. This phenomenon can further evaluate cardiac function and adjust ECMO flow in a timely fashion. Our retrospective study showed that tIPPaO_2_ is negatively correlated with patient EF and the 28-day mortality. Because VA-ECMO needs 4–6 h to routinely monitor arterial blood gas and can be performed at any time according to the changes in arterial waveform and pulse pressure difference when needed, it is a simple, easy, and cheap method with a high monitoring sensitivity and compliance. In conclusion, we believe that tIPPaO_2_ could be a good qualitative indicator for monitoring cardiac function in patients treated with VA-ECMO, which can reveal appropriate timing for adjusting VA-ECMO flow or weaning.

CA and RCS are common critical illnesses with extremely low overall survival. Reportedly, traditional CPR therapy survival rate for in-hospital CA is between 35.6 and 39.7% and between 7.7 and 8.3% for out-of-hospital CA [7, 11]. RCS mortality rate is also high at 30–60% [10] Multi-center prospective RCT studies are still lacking because multiple factors affect the VA-ECMO treatment prognosis and complex ethical issues are involved in the process. However, a few studies, such as ARREST trial [12], show that CA/RCS patients treated with VA-ECMO might improve survival compared with traditional treatment [5, 7, 11, 13]. Therefore, VA-ECMO is cautiously recommended as an alternative option for CA/RCS [1, 4, 10, 14]. The present study showed an overall survival rate similar to that in previous studies [10, 12, 15]. VA-ECMO prognosis and initiation timing for the treatment of ACF have been reported to be closely related. If the time from CA to initiation of VA-ECMO is < 30 min, the survival rate can be 50%, which is reduced to 30% at 30–60 min [7, 16, 17]. The best time for VA-ECMO support in RCS patients is before irreversible multi-organ dysfunction occurs [10, 18]. The unified criteria for the timing of initiation are currently lacking. The time from CPR to ECMO initiation in our center is ≤60 min. The RCS initiation criterion states that the dosage of vasopressor drugs continues to increase after active fluid resuscitation of > 6 h. Blood pH still progressively decreases, while lactate progressively increases, accompanied by obvious evidence of organ hypoperfusion. Whether these timings are optimal remains unclear, but we believe that if ECMO treatment is necessary after careful evaluation by a professional team, early initiation of ECMO is beneficial for the prognosis of CA/RCS patients.

VA-ECMO can be used as a bridge therapy for cardiac function recovery or definite treatment [1, 4, 5].. FMC is a self-limiting disease. The cardiac function can be gradually recovered after the outbreak period [19]. Percutaneous coronary intervention can effectively recanalize the AMI culprit vessels, providing conditions for cardiac function recovery [10, 20].. There are fewer studies on myocardial inhibition induced by other factors, where the supportive effect of VA-ECMO remains unclear [1]. The present study also found that IPPaO_2_ has a high sensitivity for predicting the prognosis of FMC and AMI, but poor sensitivity for SACM and OCCA.

According to a report, the need for CRRT adjuvant therapy in patients treated with VA-ECMO during the first 72 h is an independent risk factor for increased 90-day mortality [21]. Patients with cardiac pump failure requiring simultaneous support of VA-ECMO and CRRT usually have multiple organ dysfunction, and CRRT can effectively reduce severe fluid overload during VA-ECMO treatment [22]. A shorter tIPPaO_2_ means faster recovery of the cardiac function as shown in Table 3. Moreover, early VA-ECMO flow decline can also effectively reduce the high pressure time for CRRT and improve CRRT efficiency. The present study confirmed that CRRT time was significantly shorter in the survival group than in the non-survival group, and that tIPPaO_2_ was positively correlated with CRRT treatment time.

Heparin-induced thrombocytopenia, aggravation of primary disease, and mechanical consumption of VA-ECMO may all lead to thrombocytopenia. Studies have shown that patients treated with VA-ECMO have a significant requirement for blood transfusion, and those with an extreme need for blood transfusion have an increased mortality rate. However, transfusion-related predictors are lacking [23]. The correlation analysis indicated a negative correlation between tIPPaO_2_ and the platelet count. Thus, platelet destruction might be related to VA-ECMO flow. The specific mechanism and prediction of blood transfusion demand using tIPPaO_2_ need further experimental verification.

The present study had some limitations. First, the gold standard for assessing the “watershed” is contrast-enhanced ultrasound, which can directly identify the “watershed” location, but, on the other hand, is relatively complex. We chose bedside ultrasound as the contrast standard, because many cases of tIPPaO_2_ had occurred late at night and instant bedside ultrasound was more convenient to use. Second, the sample size of VA-ECMO was small and the occurrence of each individual disease was even lower. The present case comparison mainly focused on FMC and AMI, a small number of SACM, and cases of cardiac arrest caused by other mostly sporadic reasons. Thus, the combined OCCA treatment was chosen for analysis. The in-hospital and out-of-hospital CA was also not distinguished. Finally, this analysis was retrospective as reported in the literature. Strict and uniform randomized control criteria were not utilized and many cases of ECMO treatment options and timing were based on the clinician judgment [7, 11].

## Conclusions

In conclusion, tIPPaO2 may be a good qualitative indicator of cardiac function in patients treated with VA-ECMO that can provide a timely and reliable basis for adjusting VA-ECMO flow. tIPPaO_2_ was negatively correlated with 28-day survival probability, EF, ICU stay, length of hospitalization, and higher predictive value for acute AMI and FMC.

## Supplementary Information

**Additional file 1.**

## Data Availability

Our datasets are presented in the additional supporting files.

## Abbreviations

ACF: Acute circulatory failure
VA-ECMO: Venoarterial extracorporeal membrane oxygenation
tIPPaO2: time of inflection point of arterial oxygen partial pressure
RSC: Refractory cardiogenic shock
CA: Cardiac arrest
APACHE: Acute physiology and chronic health evaluation
CRRT: Continuous renal replacement therapy
MV: Mechanical ventilation
ICU: Intensive care unit
EF: Ejection fraction
AMI: Acute myocardial infarction
FMC: Fulminant myocarditis
SACM: Sepsis-associated cardiomyopathy
OCCA: Cardiac arrest caused by other causes
BMI: Body mass index
